# The impact of the verbal instruction and task characteristics on effect-based action control

**DOI:** 10.1007/s10339-020-00960-0

**Published:** 2020-02-21

**Authors:** Diana Vogel, Matthias Rudolf, Stefan Scherbaum

**Affiliations:** Department of Psychology, Technische Universität Dresden, 01062 Dresden, Germany

**Keywords:** Action control, Ideomotor theory, Action-effect association, Effect-based action control, Instruction, Task characteristics

## Abstract

According to ideomotor theory, when people perform a movement and observe its subsequent effect, they acquire a bidirectional action-effect association. If at a later point they want to produce the effect, its anticipation activates and allows executing the corresponding action. In ideomotor induction tasks, several task characteristics determine whether participants use the experimentally induced action-effect associations to pre-activate the corresponding actions. Here, we assess the impact of the verbal instruction, the task relevance of the effect stimuli and the presentation of post-response effects on the expression of action-effect associations. The results show that an instruction stressing the stimulus–effect correspondence prompts participants to utilize the presented effects more than an instruction stressing the stimulus-response correspondence. Furthermore, the induced action-effect associations were only expressed when the effects were relevant for the task and when post-response effects were presented in the test phase. These findings show the importance of the particular task construction for the expression of the experimentally manipulated action-effect knowledge.

## Introduction

Most of our everyday activities are goal-directed, that is, they are carried out to achieve a particular effect. This implies that we have a concept of which action leads to which effect. How do we gain this knowledge? One theory that exploits this connection is ideomotor theory (Elsner and Hommel 2001; Greenwald 1970; Harleß 1861; James 1890; for a review, see Shin et al. 2010). Ideomotor theory assumes that actions are represented in terms of their sensory consequences (i.e., their *action*-*effects*). When people execute a movement for the first time, for example in early childhood, they observe its perceivable effect and acquire a bidirectional action-effect association. If at a later time, they want to achieve the effect, they use the action-effect association “backwards” by anticipating and mentally activating the effect, which in turn triggers the corresponding action (Elsner and Hommel 2001; Janczyk et al. 2017; Kunde 2001).

Attaining goal-directed action production is a two-stage process (cf., Elsner and Hommel 2001). First, there must be the possibility to acquire action-effect associations by observing how an action is consistently followed by a particular effect on a number of occasions. Second, if the action-effect association is once established, it can be used to select the action by anticipating or recollecting its corresponding effect. Following this two-stage model, experiments on ideomotor learning usually consist of an *acquisition phase* and a *test phase*. In the acquisition phase, arbitrary actions are paired with effects. This is often obtained with free-choice tasks in which participants have to perform one of several possible actions randomly, which are then consistently followed by a particular post-response effect (e.g., left key → low tone, right key → high tone). In the subsequent *test phase*, the effect representation is activated either endogenously (e.g., when the post-response effect is expected to appear on the left after a left-sided movement) or exogenously by presenting the effect as a stimulus (e.g., a low tone is presented to trigger a left key response). In any case, the activation or anticipation of the effect is assumed to pre-activate the previously associated action. As a result, people are expected to execute the acquisition-congruent action faster than the acquisition-reversed (incongruent) action. Ideomotor experiments address this principle by implementing congruent trials, which demand the acquisition-congruent response, and incongruent trials, which demand the acquisition-reversed response. The response time (RT) difference between congruent and incongruent trials is often referred to as the *ideomotor congruency effect* and serves as an indication for the acquisition and expression of learned action-effect associations (Elsner and Hommel 2001; Hommel 1996).

Ideomotor theory assumes that the acquisition and expression of action-effect associations occur rather incidentally and automatically, even if the usage of action-effect associations is not necessary for the task (Hommel 1996, 2005; Watson et al. 2015). Phenomena relating to action planning and selection according to their corresponding sensory effects have even been measured in primates (Kuang et al. 2016). However, the ideomotor congruency effect does not appear uniformly in every setting and task setup. The verbal instruction, for instance, appears to have an effect on the performance in certain ideomotor tasks (Eder and Dignath 2017; Theeuwes et al. 2015). Also the particular design of the acquisition and the test phase modulates whether or not the experimentally induced ideomotor congruency effect is used (Herwig et al. 2007; Herwig and Waszak 2009; Pfister et al. 2011; see also Pfister 2019 for an overview). Herwig et al. (2007) claim that the task setup affects whether the system that is guiding stimulus-based actions is accompanied by stimulus–response (sensorimotor) or action-effect (ideomotor) learning. Pfister (2019), however, points out that it is less a question of whether or not ideomotor learning and the expression of action-effect associations take place at all, but rather which action-effects are used for action control. He offers a possible explanation for the inconsistently appearing ideomotor congruency effect in different studies by arguing that participants choose between different action-effects a task offers. On the one hand, these comprise the action-effect that is actually intended to be used in the experiment. These are often tones or visual effects (therefore also referred to as environment-related effects). On the other hand, people might also rely on other action-effects. If the task setup could affect which action-effect associations are used for task processing, it should also be possible to manipulate experimentally to which extent the experimentally induced action-effects are used by changing relevant task characteristics. An absent ideomotor congruency effect would indicate that participants do not use the experimentally induced action-effect associations.

The impact of task components has most often been studied using the classic ideomotor stimulus–effect congruency task, as used initially by Elsner and Hommel (2001). Here, the verbal instruction (Eder and Dignath 2017) and the design of the acquisition phase (Herwig et al. 2007) were identified as two factors to affect the acquisition and/or usage of the induced action-effect associations. Also the presentation of post-response effects appears to have an impact (Elsner and Hommel 2001). However, we argue that studying the impact of task characteristics with this task bears the risk of underestimating the effect of the task setup, because the effect stimuli are used as imperative stimuli. They require and thus automatically attract participants’ attention, which makes the task robust against effects of instruction, attention and other task characteristics. Indeed, Pfister (2019) already pointed out that the task used by Elsner and Hommel (2001) actually even seems to suggest representations outside of the experimentally induced action-effect associations. Nevertheless, the authors found an ideomotor congruency effect in most of the employed conditions, which shows the robustness of the task.

Research to date has not yet determined how these factors operate when the main task does not directly include the ideomotor information as imperative stimuli. From our point of view, a more suitable task for studying the impact of the instruction and task characteristics on action-effect associations is a basic task in which participants have to work through a simple main task, which is accompanied by previously associated ideomotor effect stimuli. If those factors have a bearing on effect-based action control, they will most likely become apparent in such a setting. The effect stimuli do not automatically attract attention and may also be neglected if not considered useful. Therefore, task characteristics are more likely to find expression. Furthermore, the use of this task to assess the impact of task characteristics provides a generalization to prior studies in understanding how secondary task factors might modulate the measured outcomes generally in ideomotor tasks.

This study therefore set out to assess the factors that determine whether experimentally induced action-effect associations are used. In order to investigate the impact of secondary task factors, we employed a simple main task which was accompanied by previously associated ideomotor effect stimuli and systematically manipulate these factors. We will present three experiments on the impact of the verbal instruction (Experiment 1), the task relevance of the effects (Experiment 2 and 3) and the presence of post-response effects (Experiment 1 and 2). In order to make a clear statement about whether these factors affect the acquisition or the usage of action-effect associations, we focused on the latter by keeping the acquisition phase constant for all conditions and all experiments. Our findings should make an important contribution to the question how the task setup is able to accentuate or suppress the usage of the induced action-effect associations in order to make deductions about the optimal setup that allows these associations to appear in the participants’ behavior.

## The impact of task characteristics on action-effect associations

From the variety of task characteristics, we focused on: (1) the verbal instruction, (2) the relevance of the effect stimuli for the main task, and (3) the presence or absence of (auditory) post-response effects. In the following, we will present a brief overview of the recent research history of these three factors.

Intuitively, it is well conceivable that verbal instructions have a strong bearing on how participants solve a task. In almost all experiments, participants have to transform a verbal instruction into an action. They are able to apply new behavior to an arbitrary task very quickly, even if they never experienced it before (Brass et al. 2009; Waszak et al. 2013). Also in the field of effect-based action control, research on the impact of instructing people either in terms of the stimulus–effect or the stimulus–response relationship is not particularly novel (Eder & Dignath 2017; Hommel 1993; Theeuwes et al. 2015; Zwosta et al. 2013). The instruction can either emphasize action-effect associations (by asking to produce an effect after a stimulus) or the response (by asking to perform a response after a stimulus), which either promotes or inhibits the expression of the induced action-effect associations. It makes sense that emphasizing the action-effect association in the verbal instruction guides the attention toward the learned relations and may therefore promote their usage. However, this was only investigated in tasks in which the effect stimuli are used as imperative stimuli in the test phase, like the classic ideomotor stimulus–effect congruency task, as used initially by Elsner and Hommel (2001). As already mentioned above, the impact of the instruction might be even smaller in this setting, because the effect stimuli automatically attract the attention and action-effect associations are stressed inevitably. The opposite is true for tasks in which the effect stimuli are solely used as additional side-stimuli. Here, the instruction has the power to explicitly draw participants’ attention toward the experimentally used action-effect associations—or even deflect it. In Experiment 1, we addressed the impact of the verbal instruction in such a setting with additional effect stimuli by employing instructions, which either stressed the stimulus–effect or the stimulus–response relationship.

The second factor, the task relevance of effect stimuli, has not been varied systematically in experiments, but naturally, different tasks imply the task relevance of effects more or less in different ways. Making the effect stimuli task-relevant can be achieved by (1) using them as imperative stimuli or different task-related signals, such as cues or go/no-go signals, or (2) stressing action-effect associations in the verbal instruction. Ideomotor congruency effects could be found with imperative or task-related effect stimuli, but also in studies in which participants were told that the effect stimuli were completely irrelevant for the test phase (Paelecke and Kunde 2007; Wolfensteller and Ruge 2011). However, the latter usually stressed the action-effect association clearly in the acquisition phase, which encourages the participants’ integration of these stimuli into their concept of the task. Seen from the perspective of the participants who can use different action-effects for action control, it would not bring much of a benefit to rely on experimentally induced action-effect associations that are not relevant for the task and do not enhance or facilitate performance. Admittedly, Hommel (1996) showed a distraction effect with irrelevant effect tones for the Simon task, but in this study, effects were always presented on the opposite side of the response (i.e., incongruent). We argue that if effect stimuli do not have any impact on the task—neither for better nor for worse—and if they are varied randomly according to their congruency to the demanded response, then participants are more likely to ignore these stimuli than when they are made task-relevant by the instruction or by employing them as task-related signals. We addressed the impact of the task relevance of effect stimuli by using them as go/no-go stimuli in Experiment 2 and compared the results with those of Experiment 1, in which the effect stimuli have no task relevance. We also controlled for these effects in Experiment 3.

For the third factor, the presentation of post-response effects in the test phase Elsner and Hommel (2001, Experiment 1B) showed that experimentally induced action-effect associations are also expressed when no post-response effects were presented in the forced-choice test phase, although the ideomotor congruency effect was significantly smaller with absent post-response effect tones. The authors explain this finding by the assumption that in congruent trials, participants are presented the same effect stimuli twice (once as a stimulus and once as a post-response effect), whereas in incongruent trials, participants are presented different effects (e.g., a low tone as effect stimulus and a high post-response effect tone). This might lead to increased confusion and longer RTs in incongruent trials and therefore falsely lead to an ideomotor congruency effect. However, a viable alternative explanation for this finding is that action-effect associations are likely to fade when they are no longer effective. When people observe a light switch not affecting the ambient light anymore, they do well to update the acquired action-effect association and not use this light switch anymore (Wirth et al. 2018). In an ideomotor experiment, this phenomenon is also to be expected. We suppose this effect to be even stronger when effect stimuli are not imperative: If actions do not lead up to the associated action-effects any longer and the usage of action-effect associations is not necessary for the task, we expect participants to be more likely to neglect the experimentally induced action-effect associations.

The impact of the presentation of post-response effects in a setting with additional effect stimuli was addressed in Experiments 1 and 2. In Experiment 1, we investigated the basic effect, with one half of the participants receiving post-response effects and the other half receiving no post-response effects. In Experiment 2, we additionally involved the possibility that the mismatch of effects in incongruent trials caused differences in the usage of action-effect associations by including a third group. Effect tones could now be either present, absent, or reversed from what the participants learned in the acquisition phase. The third group thus received post-response effects, but with mismatching effects in congruent trials.

Taken together, we conducted three experiments based on this rationale. Experiment 1 addressed the impact of the verbal instruction and the presentation of post-response effects. Experiment 2 addressed the impact of the task relevance of effect stimuli and further investigated the impact of the presentation of post-response effects. Experiment 3 controlled for a possible alternative explanation with regard to the impact of the task relevance of effect stimuli. In each experiment, participants learned the association between an action (left or right keypress) and an action-effect (low- or high-pitch tone) in the acquisition phase. This acquisition phase was analogous in all experiments, as we did not target the acquisition of action-effect associations. In the test phase, we tested whether participants’ used the acquired associations, by varying different factors across experiments. Congruency of trial was manipulated as a within-subjects factor with each participant receiving congruent and incongruent trials in a mixed manner. Effect stimuli served as additional stimuli accompanying the main task in all experiments. We tested whether the experimentally induced ideomotor action-effect associations were used, that is, whether RTs were shorter for congruent than for incongruent trials and whether the error rates were lower for congruent than for incongruent trials.

## Experiment 1

Experiment 1 aimed to investigate whether different instructions and the presentation of post-response effect tones promote or inhibit the usage of the experimentally induced action-effect associations. The experiment assessed the instruction hypothesis (Hypothesis 1) and the post-response effect hypothesis (Hypothesis 2). The instruction hypothesis (Hypothesis 1) states that the usage of the experimentally induced ideomotor action-effect associations is more likely with a stimulus–response-based (S–R, stressing the stimulus–response relationship) instruction than with a stimulus–effect based (S–E, stressing the stimulus–effect relationship) instruction. The post-response effect hypothesis (Hypothesis 2) states that the usage of the experimentally induced ideomotor action-effect associations is more likely when effect tones are presented in the test phase than when they are absent.

In the acquisition phase, participants learned the association between an action (left or right keypress) and an effect (low- or high-pitch tone). In the test phase, participants had to respond to a visual stimulus (digit) which was accompanied by one of the formerly associated post-response effect tones. With the tone and the digit, a trial could either be congruent (i.e., tone and digit related to the same response) or incongruent (i.e., tone and digit related to different responses). Participants received congruent and incongruent trials commingled.

In order to test the instruction hypothesis, participants received either an instruction emphasizing the stimulus–response key (S–R) or the stimulus–effect relationship (S–E). The S–R-based instruction stressed the stimulus–response relationship and asked the participants to press a key as response to the digit by saying: “On a digit smaller than five press the left key.” The S–E-based instruction stressed the stimulus–effect relationship by saying: “On a digit smaller than five produce a low-pitch tone.” We expected the instruction to affect task encoding: With the S–R-based instruction, it should be less likely that participants use the experimentally induced action-effect associations in order to activate the response than with an S–E-based instruction.

In order to test the post-response effect hypothesis (Hypothesis 2), one-half of the participants of each group was presented post-response effect tones after each keypress, the other half was presented no post-response tones.^1^

### Methods

#### Participants

Eighty undergraduate students from the University of Dresden (mean age = 23.4 years, 59 female, 40 with S–R-based instruction, 40 with S–E-based instruction) performed the experiment.^2^ All participants had normal or corrected-to-normal vision and were naive regarding the hypotheses underlying the experiment. One participant had to be replaced for pressing one key in 80% of the trials of the learning phase. This resulted in a final sample of 40 participants in each condition with a left key percentage between 35 and 65% in the acquisition phase.

#### Apparatus and stimuli

The auditory effect stimuli were sinusoidal tones of 440 or 880 Hz lasting for 200 ms and were presented via headphones. Visual stimuli were presented in white against a black background on a 17-inch CRT screen with a resolution of 1280 × 1024 pixels. Imperative stimuli were digits from 1 to 9, excluding 5. Responses were carried out via the left and right control key on a standard computer keyboard using the index finger of the left or right hand. As control software, we used MATLAB 2010 (Sharma and Martin 2009) and Psychophysics Toolbox 3 (Brainard 1997; Kleiner et al. 2007) on a Windows 7 computer.

#### Procedure

The experiment was divided into an acquisition phase and a test phase.

##### Acquisition phase

Each trial started with a white fixation cross. Participants had to respond to this fixation cross by pressing the left or the right key as quickly as possible. They were instructed to choose freely which key to press, to use the keys in a random order, avoid response patterns, and to use each key about equally often. Each keypress triggered the presentation of a post-response effect tone. For half of the participants, the left key was followed by a low-pitch tone and the right key was followed by a high-pitch tone. For the other half, the action-effect mapping was reversed. Participants were told that the tones, which followed their keypresses, were irrelevant for the task. The trial ended after the post-response effect tone or after a deadline of 1.5 s. If the deadline was missed, written feedback was displayed (“Too slow reaction” in German language) and the next trial started after an inter-trial interval of 1.5 s by displaying the fixation cross. Participants worked through 16 practice trials and 4 blocks with 50 acquisition trials per block, resulting in 200 acquisition trials. After each block, they were allowed to take a short break.

##### Test phase

Each trial started with a white fixation cross appearing for 500 ms. After the cross disappeared, the white digit was presented in the middle of the screen. Regardless of what the instruction said in detail, participants always had to press the left key for digits smaller than 5 and the right key for digits greater than 5 as fast as possible. Simultaneously to the digit, one of the two effect tones already employed in the acquisition phase was presented. With the digit and the tone, each trial could be either acquisition-congruent or incongruent. Participants were randomly assigned to two groups: The first group received the S–R-based instruction, the second group received the S–E-based instruction. The two instructions resulted in the same behavioral action.

Each group of participants was further divided into two groups: For half of the participants of each group, each keypress triggered the presentation of a post-response effect tone. The response-effect mapping remained unchanged from the acquisition phase for this group. The other half did not hear post-response effect tones after a keypress.

Participants worked through 20 practice trials and 4 blocks with 48 trials each block, resulting in 192 test trials. After each block, they were allowed to take a short break. Data analysis was conducted using SPSS version 25 (IBM Corp. 2017) and R (R Development Core Team 2018).

### Results

#### Acquisition phase

Trials with response omission (0.19%) were excluded from the analyses. A *t* test yielded that the response ratio difference from 50% reached significance (49.0% vs. 51.0%, *t*(79) = 2.49, *p* = .015, *d* = 0.56). Nevertheless, it can be assumed that participants experienced both R-E couplings often enough to allow for ideomotor learning. RTs did not differ between left and right responses (*M*_left_ = 219 ms, *M*_right_ = 222 ms, SD_left_ = 69 ms, SD_right_ = 66 ms), *t*(79) = 1.22, *p* = .226, *d* = 0.28.

#### Test phase

Trials with response omissions (0.18%) were excluded from all following analyses. The instruction hypothesis stated that the ideomotor congruency effect is smaller with an S–R based instruction than with an S–E-based instruction. The post-response effect hypothesis stated that the RT congruency effect is greater with effect tones than without effect tones. In order to test these two hypotheses, we conducted a mixed-design analysis of variance (ANOVA) with RTs as dependent variable. Congruency of trial was included as repeated measures variable. Instruction (S–R or S–E) and the presentation of post-response effect tones (presented or not) were included as independent group factors.

Table 1 shows the descriptive statistics of the response time data and the error rates. The analysis revealed the following results.

**Table 1:** Descriptive statistics of RTs and error rates in Experiment 1 for all experimental conditions

	Response times in ms			
	Error rates in %			
	S–R instruction		S–E instruction	
	*M*	SD	*M*	SD
Effect tones presented				
Congruent	408	37	413	42
	2.86	2.55	2.69	2.04
Incongruent	412	32	424	43
	3.91	3.11	3.54	3.30
No effect tones presented				
Congruent	428	38	424	41
	2.97	2.83	3.22	2.23
Incongruent	428	37	429	39
	3.33	3.51	3.91	2.62

A main effect of congruency shows that RTs in congruent trials were shorter than in incongruent trials, *F*(1,76) = 16.47, *p* < .001, $\eta^{2}_{p}$ = 0.18. This difference is larger with the S–E-based instruction than with the S–R instruction, as indicated by an interaction of congruency and instruction, *F*(1,76) = 6.03, *p* = .016, $\eta^{2}_{p}$ = 0.73. Post hoc analyses showed that the ideomotor RT congruency effect was indeed only significant with the S–E-based instruction, *t*(39) = 5.22, *p* < .001, *d* = 1.67, but not with the S–R-based instruction, *t*(39) = 1.00, *p* = .326, *d* = 0.32. The analysis also revealed an interaction of congruency and post-response effect tone presentation, *F*(1,76) = 4.95, *p* = .029, $\eta^{2}_{p}$ = .61, indicating that participants who were presented post-response effect tones showed a greater ideomotor congruency effect. The three-way interaction of congruency, instruction, and post-response effect tone presentation was not significant, *F*(1,76) = 0.09, *p* = .768, $\eta^{2}_{p}$ < .01, showing that the impact of the instruction and the post-response effect tones are independent for the congruency effect. This finding was also confirmed by a Bayesian mixed-design ANOVA, which yielded a moderate evidence in favor of the H_0_ for the three-way interaction, BF_10_ = 0.302. Neither the main effect of instruction nor the main effect of post-response effect tone presentation nor the interaction between these two variables reached significance (all *F*_s_ < 2.32).

To address error rates, we ran a mixed-design ANOVA with error rates as dependent variable. Instruction (S–R or S–E) and the presentation of post-response effect tones (presented or not) were included as independent group factors. A main effect of congruency showed that participants made more errors in incongruent than in congruent trials, *F*(1,76) = 5.24, *p* = .025, $\eta^{2}_{p}$ = .06. No other effect reached significance (all *F*_s_ < 1).

### Discussion

The first hypothesis we aimed to assess with Experiment 1 was the instruction hypothesis that an S–E-based instruction leads to a stronger expression of the experimentally induced action-effect associations than an S–R-based instruction. The results of the experiment confirm this hypothesis. We found a larger ideomotor congruency effect for the S–E-based instruction, as compared to the S–R-based instruction. The S–R-based instruction appears to suppress the usage of the experimentally induced action-effect associations due to the emphasis of the response (“press the left key”) rather than action-effects. In contrast, the S–E-based instruction puts more emphasis on the induced effects, which appears to promote the usage of the experimentally induced action-effect associations. This is consistent with the findings of Eder and Dignath (2017) who also reported a larger ideomotor congruency effect when the instruction asked the participants to produce effect tones intentionally in the Elsner and Hommel (2001) task. However, the ideomotor congruency effect we found in our experiment turned out to be much smaller (between 4 and 11 ms, as compared to about 100 ms in the intention instruction in the study of Eder and Dignath (2017). Two conceivable explanations for this discrepancy follow directly from the design of the study. First, in our task, the effect stimuli were not imperative; hence, the impact of the experimentally induced action-effect associations might be more unstable. Second, in the study by Eder and Dignath, congruency was implemented as a group factor whereas it was varied trial-wise in our study. It makes sense that keeping a constant mapping would yield a greater ideomotor congruency effect than a trial-wise manipulation. Indeed, it is even more encouraging that the expression of action-effect associations appears in both experimental settings and that task features like the verbal instruction operate in a similar manner.

The second hypothesis we aimed to asses with Experiment 1 was the post-response effect hypothesis that the presentation of post-response effects leads to a stronger expression of the experimentally induced action-effect associations than their absence. The results of Experiment 1 are also in line with this hypothesis. Intuitively, it seems conceivable that the actual presentation of action-effects makes participants use the corresponding action-effect associations more likely. However, this outcome is contrary to those from Elsner and Hommel (2001) who found an ideomotor congruency effect even in the group who heard no post-response effect tones in the test phase—albeit smaller than with post-response effect tones. This might be due to the amount of attention the participants draw to the experimentally induced action-effect associations. In our study, participants generally paid less attention to the effect stimuli, because they were not relevant for the task. In the study of Elsner and Hommel (2001), it was not possible to withdraw the attention from the (imperative) effect stimuli while working through the task, making the action-effect associations more salient. Additionally, as mentioned above, congruency was varied as a within-subjects factor in our study, which might also yield weaker congruency effects.

Taken together, Experiment 1 identified two factors to affect to which extend the experimentally induced action-effect associations are used for handling the task. These are the verbal instruction and the presentation of post-response effects. However, it still leaves the question open whether the impact of the post-response effects originates from the fact that incongruent trials always bear two mismatching tones and therefore irritate the participants (as suggested by Elsner and Hommel 2001), or from the fact that they render the actual action-effect association more salient. In order to gain insight into this, Experiment 2 further investigates the impact of post-response effects. Here, one group of participants received post-response effects, the second group received acquisition-reversed post-response effects and the third group received no post-response effects. If this effect were driven by the mismatch of tones, the first and second groups would express the experimentally induced ideomotor congruency effect while the third group would express no effect.

Furthermore, Experiment 2 seeks to address another factor to have an impact on the expression of the experimentally induced action-effect associations, namely the task relevance of effects.

## Experiment 2

There are two primary aims of Experiment 2: First, we aimed to investigate the task relevance hypothesis (Hypothesis 3) that the usage of the experimentally induced ideomotor action-effect associations is more likely when the effect stimuli are task-relevant. Therefore, Experiment 2 was similar to Experiment 1 with the S–R-based instruction, but with an additional go/no-go task that made the effect stimuli task-relevant.

In Experiment 1, several participants reported suppressing to listen to the auditory material in order to avoid distraction from the main task. This made us suggest that participants primarily use information that creates benefit for their intended goals. If information is no longer necessary for the task, it gets more and more neglected. In other words, participants use the experimentally induced action-effect associations more often when it creates any kind of benefit. Therefore, we expect the participants to be more likely to express experimentally induced response-effect associations if the effect tones are relevant for the task. To this end, we used the paradigm of Experiment 1, but added a go/no-go task to the test phase to change the task relevance of the effect stimuli. Here, participants were presented one of *three* different tones while a digit was displayed. This tone could be either one of the two former effect tones or a third tone with a pitch between the two effect tones. The former effect tones served as go signals and the third tone served as a no-go signal. We used the S–R instruction of Experiment 1 in order to keep the experimental conditions parallel in terms of the instruction.

Second, for a further investigation of the post-response effect hypothesis (Hypothesis 2), we aimed to ascertain whether the mechanism driving the impact of the post-response effect tone presentation is the mismatch of effect stimuli in incongruent trials. Therefore, participants were presented post-response effect tones in an acquisition-congruent mapping, in an acquisition-reversed mapping, or they were presented with no post-response effect tones in the test phase. If the ideomotor congruency effect were solely dependent of the matching of tones, RTs of congruent and incongruent trials would differ in the test phase with the acquisition-congruent response-effect mapping as well as with the acquisition-reversed mapping. If the ideomotor congruency effect were dependent on the usage of action-effect associations in the acquisition phase, RTs of congruent and incongruent trials would differ with the acquisition-congruent response-effect mapping, but not with the acquisition-reversed mapping.

### Methods

#### Participants

Sixty undergraduate students from the University of Dresden (mean age = 23.9 years, 50 female, *N*_present_ = 20, *N*_absent_ = 20, *N*_reversed_ = 20) performed the experiment.^3^ All participants had normal or corrected-to-normal vision and were naive regarding the hypotheses underlying the experiment. One participant had to be excluded from the analyses for pressing exclusively the right key in the acquisition phase. Five participants had to be excluded from the analyses because of error rates greater than 85% in the go/no-go task. They were replaced in random order by another six participants. This resulted in a final sample of 20 participants in each condition with a left key percentage between 40 and 60% in the acquisition phase and an error rate in the go/no-go task under 50%.

#### Apparatus and stimuli

Stimuli and setup followed those of Experiment 1 with one exception: Auditory stimuli were sinusoidal tones of 440, 660 or 880 Hz lasting 200 ms.

#### Procedure

The experiment was divided into an acquisition phase and a test phase.

##### Acquisition phase

The trial procedure of the acquisition phase was analogous to Experiment 1.

##### Test phase

The trial procedure followed the logic of Experiment 1 with two major changes. First, only the stimulus-based instruction of Experiment 1 was used, which asked the participants to press a certain key after seeing a certain digit. Second, in contrast to Experiment 1, the tone that was presented simultaneously with the digit was either one of the two effect tones already employed in the acquisition phase (the low- and the high-pitch tone) or another tone with a pitch lying between the other tones (the middle-pitch tone). With the middle-pitch tone, a go/no-go task was introduced. The low-pitch and the high-pitch tones served as go stimuli and the middle-pitch tone served as no-go stimulus. In go trials, participants had to react to the digit just as in Experiment 1. In no-go trials, they were instructed not to press any key. Third, participants were randomly assigned to one of three groups that experienced different post-response effect tones in the test phase. The first group was presented post-response effect tones after each valid go-response with unchanged response-effect mapping from the acquisition phase. The second group was also presented post-response effect tones after each valid go-response, but the action-effect mapping was reversed from the acquisition phase. The third group was never presented any effect tones in the test phase. In no-go trials, no effect tone was presented for each group. Participants worked through 20 practice trials and 4 blocks with 48 trials each block, resulting in 192 test trials. The practice trials started with one go trial employing the low-pitch tone followed by one go trial employing the high-pitch tone to ensure that the participants classify the low, middle and high tones appropriately. The trials of each block consisted of 2/3 go trials and 1/3 no-go trials in a randomized order. Congruency of trial was included as a within-subjects factor. Participants received congruent and incongruent trials commingled in the go trials. After each block, participants were allowed to take a short break.

### Results

#### Acquisition phase

Trials with response omission (0.15%) were excluded from the analyses. A *t* test yielded that response ratio did not differ from 50% (49.4% vs. 50.6%, *t*(59) = 1.83, *p* = .073, *d* = 0.48); thus, participants experienced both R–E couplings about equally often. RTs did not differ between left and right responses (*M*_left_ = 208 ms, *M*_right_ = 214 ms, SD_left_ = 69 ms, SD_right_ = 65 ms), *t*(59) = 1.74, *p* = .087, *d* = 0.45.

#### Test phase

Go-trials with response omissions (2.0%) were excluded from all following analyses. To address the question if task relevance promotes the usage of learned action-effect associations, we ran a mixed-design ANOVA with RT as dependent variable. Congruency of trial was included as repeated measures variable. The presentation of post-response effect tones (with effect tones, without effect tones and with reversed effect tones) was included as independent group factor. Table 2 shows the descriptive statistics of the RT data.

**Table 2:** Descriptive statistics of RTs in Experiment 2 for all experimental conditions

	Response times in ms	
	*M*	SD
Effect tones presented		
Congruent	540	127
incongruent	585	145
Effect tones reversed		
Congruent	535	128
Incongruent	509	93
No effect tones presented		
Congruent	570	126
Incongruent	558	114

The analysis revealed no main effect of congruency over all groups, *F*(1,57) = 0.08, *p* = .778, $\eta^{2}_{p}$ < .01, but an interaction of congruency and post-response effect tones, *F*(2,57) = 7.53, *p* = .001, $\eta^{2}_{p}$ = .21. The main effect of post-response effect tones was not significant, *F*(2,57) = 0.79, *p* = .459, $\eta^{2}_{p}$ = .02. To assess the impact of post-response effect tones in more detail, we ran paired samples *t* tests for the three groups separately and compared RTs for congruent and incongruent trials. For the group with acquisition-congruent post-response effect tones, the *t* test revealed that RTs in congruent trials were shorter than in incongruent trials, *t*(19) = 4.29, *p* < .001, *d* = 1.96. However, this was not true for the group that heard acquisition-reversed post-response effect tones, *t*(19) = 1.5, *p* = .140, *d* = 0.68, or no post-response effect tones, *t*(19) = 0.93, *p* = .365, *d* = 0.43.

To address error rates of go trials, we ran another mixed-design ANOVA with error rates as dependent variable. Congruency of trial was included as repeated measures variable. The presentation of post-response effect tones (with effect tones, without effect tones and with reversed effect tones) was included as independent group factor (see Table 3 for the descriptive statistics). The analysis yielded no significant main effect of congruency, *F*(1,57) = 1.19, *p* = .280, $\eta^{2}_{p}$ = .20, or post-response effect tones, *F*(1,57) = 0.98, *p* = .381, $\eta^{2}_{p}$ = .03, but a significant interaction of congruency and effect tones, *F*(2,57) = 11.03, *p* < .001, $\eta^{2}_{p}$ = .28. Post-hoc analyses showed that participants in the group with acquisition-congruent post-response effect tones made more errors in congruent than in incongruent trials, *t*(19) = 5.18, *p* < .001, *d* = 2.38. This was not true for the group with acquisition-reversed post-response effect tones, *t*(19) = 1.10, *p* = .284, *d* = 0.50, or no post-response effect tones, *t*(19) = 1.30, *p* = .208, *d* = 0.60. Altogether, the error rates also reflect the response time patterns.

**Table 3:** Descriptive statistics of error rates in Experiment 2 for congruency and presentation of post-response effects

	Error rates in %	
	*M*	SD
Effect tones presented		
Congruent	5.55	5.03
Incongruent	16.41	7.80
Effect tones reversed		
Congruent	11.17	6.48
Incongruent	8.67	6.66
No effect tones presented		
Congruent	14.06	11.05
Incongruent	10.31	6.42

To compare error rates between go and no-go trials, we ran another mixed-design ANOVA with error rates as dependent variable, go/no-go condition as repeated measures variable and the presentation of post-response effect tones (with effect tones, without effect tones and with reversed effect tones) as independent group factor (see Table 4 for the descriptive statistics). Over all groups, participants made more errors in no-go trials than in go trials, as indicated by a main effect of go/no-go, *F*(1,57) = 11.23, *p* = .001, $\eta^{2}_{p}$ = .17, but there was no interaction of go/no-go and post-response effect tones, *F*(2,57) = 1.81, *p* = .172, $\eta^{2}_{p}$ = .06.

**Table 4:** Descriptive statistics of error rates in Experiment 2 for go and no-go trials

Trial	Error rates in %	
	*M*	SD
Go	11.03	5.11
No-go	14.71	9.41

**Fig. 1:**
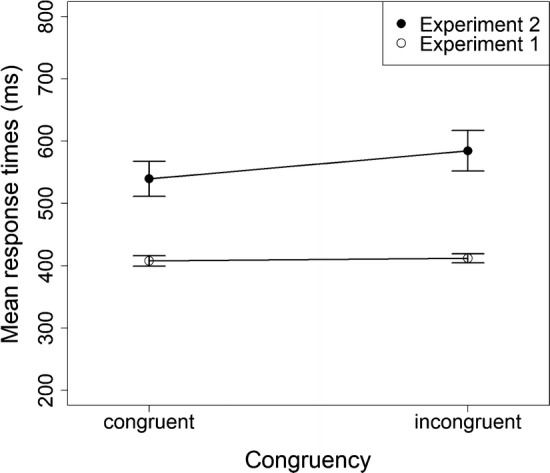
Mean response times of congruent and incongruent trials in the test phase of Experiment 1 (only S–R based instruction and effect tones) and Experiment 2 (with acquisition-congruent effect tones). The ideomotor congruency effect is only visible in Experiment 2 that includes the no-go task and therefore renders action-effects task-relevant

In order to gain insight into the impact of the task relevance of post-response effect tones on the expression of action-effect associations, we ran another analysis and combined the data of the group with acquisition-congruent post-response effect tones in Experiment 2 and the group that experienced the S–R-based instruction and presented post-response effect tones in Experiment 1. Hence, experimental conditions for these two groups are analogous except for the go/no-go task for the group of Experiment 2. We conducted a mixed-design ANOVA for this combined sample with RT as dependent variable. Congruency was included as a within-subjects factor, and Experiment (1 or 2) was included as an independent group factor. The analysis revealed a main effect of congruency, *F*(1,38) = 20.74, *p* < .001, $\eta^{2}_{p}$ = .35, showing that the participants had shorter RTs in congruent trials than in incongruent trials. A main effect of experiment showed that the participants responded more slowly in the Experiment 2, which included the go/no-go task, *F*(1,38) = 24.18, *p* < .001, $\eta^{2}_{p}$ = .39. An interaction of congruency and experiment showed that the ideomotor congruency effect was stronger in Experiment 2 than in Experiment 1, *F*(1,38) = 14.27, *p* = .001, $\eta^{2}_{p}$ = .27 (see Fig. 1).

### Discussion

Experiment 2 aimed to assess the task relevance hypothesis (Hypothesis 3) assuming that rendering the effect tones task-relevant leads to a stronger expression of the experimentally induced action-effect associations. For the group who heard acquisition-congruent post-response effect tones, this could be confirmed. Participants in this group showed an ideomotor congruency effect and thus appeared to use the previously acquired action-effect associations more than the analogous group in Experiment 1 who experienced task-irrelevant effect stimuli.

Regarding the impact of post-response effects, we found a similar pattern as in Experiment 1: The group with acquisition-congruent post-response effect tones expressed the experimentally induced action-effect associations. The group with absent post-response effect tones did not show this effect. Beyond that, in Experiment 2, we found that the group with acquisition-reversed post-response effect tones did not show an ideomotor congruency effect. These findings dispels the possibility that in the group with acquisition-congruent post-response effect tones, the ideomotor congruency effect appeared because participants heard two mismatching tones in the incongruent trials and therefore respond more cautiously. If this were the case, the group experiencing acquisition-reversed post-response effect tones should have also shown an ideomotor congruency effect. Our results thus rather suggest that post-response effects play a part in contributing to making the experimentally induced action-effect associations more salient and therefore uphold the associations.

In summary, Experiment 2 identified the task relevance of effect tones as another factor to affect whether the experimentally induced action-effects are used for handling the task. Furthermore, the presentation of post-response effects appears to uphold the experimentally induced action-effects associations in a setting with subsidiary effect presentations.

However, Experiment 2 still leaves one source of for a potential alternative explanation for its results. This is the question whether or not the introduction of the go/no-go task did not only render the effect stimuli task-relevant but also changed the whole task procedure. It is conceivable that the mere introduction of the go/no-go task changed the way participants work through the task, for instance by raising the amount of cognitive control needed for the task, which could eventually lead to a greater ideomotor congruency effect. Ruling out this possible confound required another experiment that employs the same task combination with the digit task and the go/no-go task, but with task-irrelevant effect stimuli. This was realized in Experiment 3.

## Experiment 3

Experiment 3 was an AsPredicted preregistered study (https://osf.io/s52zu/) that further explored the role of the task relevance of effect stimuli (Hypothesis 3). It employed a visual go/no-go task to control for the possible confound in Experiment 2 that the introduction of the go/no-go task itself—not the task relevance of the effect tones—influenced the expression of action-effect associations. Experiment 3 thus involved a go/no-go task without making the effect stimuli relevant for the task. If the expression of action-effect associations were only triggered by the go/no-go task, there would be RT differences between congruent and incongruent trials in Experiment 3. If action-effect associations were expressed because the effect stimuli were made task relevant, then there would be no RT differences between congruent and incongruent choices in Experiment 3. The main hypothesis of Experiment 3 is, with a visual no-go setting but no task-relevant effect stimuli, participants will not show an ideomotor congruency effect.

### Methods

#### Participants

According to the preregistration plan, 25 undergraduate students from the University of Dresden (mean age = 25.4 years, 20 female) performed the experiment.^4^ All participants had normal or corrected-to-normal vision and were naive regarding the hypotheses underlying the experiment.

#### Apparatus and stimuli

Equipment details were as in experiments 1 and 2.

#### Procedure

The experiment was divided into an acquisition and a test phase.

##### Acquisition phase

The trial procedure of the acquisition phase was the same as in experiments 1 and 2.

##### Test phase

The trial procedure was analogous to Experiment 2 with one exception: The go/no-go task was implemented using a visual instead of auditory stimulus. The no-go stimulus was a dark gray digit background. When the presented digit was surrounded by the dark gray background, participants were instructed not to press any key.

### Results

#### Acquisition phase

Trials with response omission (0.18%) were excluded from the analyses. A *t* test yielded that response ratio did not differ from 50% (49.3% vs. 50.7%, *t*(24) = 1.00, *p* = .327, *d* = 0.41); thus, participants experienced both R–E couplings about equally often. RTs did not differ between left and right responses (*M*_left_ = 215 ms, *M*_right_ = 221 ms, SD_left_ = 82 ms, SD_right_ = 81 ms), *t*(24) = 1.25, *p* = .225, *d* = 0.51.

#### Test phase

Go trials with response omissions (0.6%) were excluded from all following analyses. The analysis was conducted according to the preregistration plan. To address the hypothesis that there is no ideomotor congruency effect when a visual go/no-go task is employed, we ran a paired samples *t* test that revealed no RT difference between congruent and incongruent trials (*M*_congruent_ = 457 ms, *M*_incongruent_ = 459 ms, SD_congruent_ = 56 ms, SD_incongruent_ = 62 ms), *t*(24) = 0.90, *p* = .378, *d* = 0.36. As the *t* test is not a sufficient analysis for detecting the absence of an effect in mean differences, we also ran a paired-samples “two-one-sided *t* tests” (TOST) procedure for equivalence testing. As the effect size of for the ideomotor congruency effect was *d* = − 1.96 in Experiment 2, we set the smallest effect size of interest to *d* = ± 0.6. The TOST analysis was conducted using the TOSTER package for R (Lakens 2017). The equivalence test was significant, *t*(24) = 2.10, *p* = .023, *d* = 0.86. Based on the equivalence test and the null-hypothesis *t* test combined, it can be concluded that the observed effect is statistically not different from zero and statistically equivalent to zero. In addition, a Bayesian paired samples *t* test yielded a moderate evidence in favor of the *H*_0_, BF_10_ = 0.304 (following the classification suggested by Jeffreys 1961).

Regarding error rates, a paired samples *t* test showed that surprisingly, participants made more errors in go than in no-go trials, *t*(24) = 2.32, *p* = .030, *d* = 0.95. They also made more errors in congruent than in incongruent trials, *t*(24) = 2.25, *p* = .034, *d* = 0.92.

### Discussion

Experiment 3 set out with the aim of addressing the possible confound that the expression of action-effect associations in the group with acquisition-congruent effect tones in Experiment 2 could only be ascribed to the introduction of the go/no-go task and not to the task relevance of effect stimuli. The results of Experiment 3 reject these concerns. Employing a visual go/no-go task simultaneously to the digit task with task-irrelevant effect tones did not lead to the expression of an ideomotor congruency effect for the experimentally induced action-effect associations. Experiment 3 thus confirms that the task relevance of effect tones—and not the task itself—determines whether the experimentally induced action-effect associations are used.

At first glance, the error rate patterns appear odd in this experiment. Participants made more errors in congruent than in incongruent trials. However, this finding can be seen as a further indication that participants did not use the experimentally induced action-effect associations for this setting. For the finding that participants made more errors in go than in no-go trials, a possible explanation is that in no-go trials, the stimuli had a gray background. This could have rendered the no-go stimuli more salient, which lead to increased attention and therefore decreased error rates.

## General discussion

We assessed the impact of verbal instruction and task features on the expression of the experimentally induced action-effect associations in three experiments. We varied the verbal instruction, the presentation of post-response effects and the task relevance of effect stimuli. We hypothesized that participants are more likely to express the experimentally induced action-effect associations (1) with an S–E-based instruction rather than with an S–R-based instruction, (2) when they are presented post-response effect tones in the test phase, and (3) when the effect stimuli are task-relevant. Our results mainly support these three assumptions. However, they also indicate that these components are highly interconnected and their impact cannot be assessed independently.

### The role of the verbal instruction

Experiment 1 showed that an S–E-based instruction promotes the expression of experimentally induced action-effect associations more than an S–R-based instruction. This finding supports the results of earlier studies (Eder and Dignath 2017; Theeuwes et al. 2015; Zwosta et al. 2013). In other words, participants are more likely to make use of the induced action-effect associations when they are stressed by the instruction. An instruction stressing the response, in contrast, appears to impede the usage of these action-effect associations. However, the effect sizes we found for the ideomotor congruency effect for the different instructions are rather small, as compared to the work of Eder and Dignath (2017) for example, who used the traditional paradigm proposed by Elsner and Hommel (2001). Aside from that, Elsner and Hommel (2001) still found an ideomotor congruency effect of the experimentally induced action-effect associations while using an S–R-based instruction, whereas it was absent in similar conditions in our study. The impact of the verbal instruction seems to be highly interconnected with the respective task setting that is used. With imperative effect stimuli, it might be less important to stress experimentally induced action-effect associations explicitly in the instruction than in a setting in which effect stimuli only accompany the imperative stimuli. Thus, based on our results, we recommend seeing the verbal instruction always in context with the actual task.

The interaction of instruction and congruency did show in the RTs, but not in the error rates. Here, only an effect of congruency showed, indicating that participants made more errors in incongruent trials than in congruent trials. This could be attributed to the generally low effect sizes. In this setting, the impact of the instruction was not strong enough to find expression in the error rates.

On closer inspection of the data of Experiment 1, it might appear odd that the ideomotor congruency effect also appeared in the group that received an R–E-based instruction without post-response effect tones in the test phase. These participants were asked to produce a certain tone even though this tone did not follow the keypress in the test phase. Pfister et al. (2014) offered an explanation for this finding by reporting the incorporation of action-effect associations into action control even if the effect is never actually experienced. It might be that specific action-effect knowledge originating from the S–E-based instruction prompts an internal anticipation of effects, which is used for action control without depending on the actual presentation of effect tones.

In summary, the verbal instruction is able to affect whether participants use the experimentally induced action-effect associations for a task. An S–E-based instruction promotes the usage of these action-effect associations, while an S–R-based instruction appears to impede their usage.

### The role of the task relevance of effect stimuli and the presentation of post-response effects

We identified the task relevance of effect stimuli and the presentation of post-response effects as two task characteristics to affect whether the experimentally induced action-effect associations are used. The results of Experiment 2 showed that task-relevant effect stimuli are more likely to be used for action control than effect stimuli that do not help the participants managing the main task. Experiment 3 also confirmed that this relation is not reducible to the very task that is employed. Hence, participants are more likely to rely on the experimentally induced action-effect associations when it is helpful for achieving their goals. In contrast, they are more likely to rely on other action-effect associations when the induced effects are only used as a (potentially interfering) side factor. This suggests that people are able to choose the effect representation according to their intended goals and therefore work efficiently through tasks.

Nevertheless, both the instruction and the task relevance of effect stimuli only trigger the expression of the experimentally induced action-effect associations when acquisition-congruent post-response effects are presented. In both experiments 1 and 2, only the group who heard acquisition-congruent post-response effect tones in the test phase showed an ideomotor congruency effect. This finding, which differs from the results found by Elsner and Hommel (2001), is most likely to explain by the nature of the task we used in our study. The main task of experiments 1 and 2 was a simple digit task and participants mainly should have pursued the goal in order to solve the task correctly. Strictly speaking, for Experiment 1, the effect stimuli did not really play a role for the objective of solving the digit task, especially with the S–R-based instruction, which did merely ask to press a key after hearing a digit. In principle, it was even possible to work through the task without paying attention to the effect stimuli at all and that would not even be the worst strategy for the task. In Experiment 2, participants only had to pay attention to the middle tone that announced a no-go trial. Otherwise, the tones were not advantageous. Hence, the effect stimuli were much less valuable than in a task with imperative effect stimuli. Thus, action-effect associations could likely been neglected in this setting. The presentation of post-response effects counteracts this negligence by constantly presenting the acquired action-effect associations and consequently refreshes them permanently. This might be the reason why the presentation of post-response effects played such a crucial role in our study, whereas it was less important in other studies (Herwig et al. 2007; Paelecke and Kunde 2007; see also Pfister et al. 2014 for a setting with unperceivable effects).

In summary, our results suggest that in a setting in which effect stimuli are not used as imperative stimuli, participants are more likely to use the experimentally induced action-effect associations when the effect stimuli are task-relevant and when post-response effects are presented.

### General considerations and limitations

Generally speaking, ideomotor theory claims that finding an ideomotor congruency effect needs two processes to take place: the acquisition and the usage of action-effect associations. Hence, when one fails to find an ideomotor congruency effect it is difficult to attribute this missing effect to one or the other process. From our point of view, the usage of action-effect associations might be the more likely reason for a congruency effect to fail to appear. In our study, we kept the acquisition phase constant to make sure that learning is the same for each participant and each experiment. Hence, the results of our study are indicative for a (non-)usage of action-effect associations and not for failures in their acquisition.

A potential point of criticism could be that in each of our experiments, we used a fixed presentation interval for the fixation cross. Thus, the participants were able to predict the occurrence of the imperative stimulus temporally. This factor is also known to affect (effect-based) action control in some settings (e.g., Ruess et al. 2018; Thomaschke and Dreisbach 2013). By holding this factor constant, we made sure that temporal predictability of the stimuli is no alternative explanation for our findings. As the non-predictability of an imperative stimulus would make the main task more difficult in our setting, it is possible that participants use the effect stimuli and therefore the induced action-effect associations more than in a predictable setting like our experiments. Thus, in order to develop a comprehensive list of the factors to affect whether the experimentally induced action-effect associations are used, temporal predictability might be a promising factor to consider in further research.

What we could not control for, even though we investigated the impact of different experimental features, was participants’ theory on what was measured during the task. This is of course the case in most ideomotor experiments, but in our study, this could serve as a possible confound. Some participants reported assuming the experiment to measure their attention. According to their theory, the tones served as distractors, which they had to inhibit. This might be another factor to affect whether the experimentally induced action-effect associations are used for the task. Indeed, it is conceivable that participants rely more on other action-effect associations when the experimentally induced action-effects distract from their actual goal of managing the main task. In the task setting we used for our study, the task characteristics and the participants’ theory on the study’s goal are hard to tell apart. To determine the exact impact of the participants’ experiment theory, it needs further studies, which allow a clear statement about this issue.

### Practical implications

Ideomotor learning and effect-based action control are two well-studied factors in human action control (Hommel et al. 2016). In principle, they are suitable for psychological studies for they are believed to take place incidentally, implicitly and automatically (Watson et al. 2015). However, beyond the experimentally induced action-effects, experiment-independent action-effects, for instance body-related action-effects, are still sufficient for ideomotor action control (Pfister 2019). We showed that several task characteristics might hamper the measurement of ideomotor learning and effect-based action control in research settings as they impede the usage of the experimentally induced action-effects. This implies the possibility of not finding ideomotor congruency effects in experiments solely because of the impact of these “secondary” task factors and therefore bearing the risk of false-negative outcomes. In order to prevent jumping to conclusions in ideomotor studies, we thus consider it necessary to employ these factors in ideomotor experiments with deliberation according to the study’s objective.

The results of our study suggest (1) employing a verbal instruction that stresses the action-effect associations induced in the experiment, (2) employing task-relevant effect stimuli from which participants benefit for the main task, (3) employing acquisition-congruent post-response effects in the test phase to prevent negligence of the action-effect associations in settings with temporal-predictable conditions. Effect stimuli can be employed as imperative stimuli or as an addition to a distinct main task; ideomotor learning and the expression of action-effect associations can proceed in either case. Beyond that, Watson et al. (2015) recommend using a small set of action-effect associations. Herbort and Butz (2012) support this claim and adds that effects should follow the action within a short time window and the demanded actions have to be rather simple.

## Conclusion

Our study has shown that the verbal instruction, the task relevance of effect stimuli and the presentation of post-response effects in the test phase affect whether the experimentally induced action-effect associations are used for working through a task. The more a task stresses the induced action-effect associations and the more they contribute to a good performance in the task, the more likely participants are going to use these associations. In this sense, in addition to the design of the acquisition phase (Herwig et al. 2007), the complexity of the task (Watson et al. 2015) and other factors, the task relevance of effect tones and the presentation of post-response effects are two more factors to affect whether the induced action-effect associations are used for action control.

## Data Availability

All data, analysis scripts and outputs have been made publicly available via the Open Science Framework (OSF) and can be accessed at https://osf.io/uxfz2/. Materials for this study have not been made publicly available. The design and analysis plan for Experiment 3 were preregistered on the OSF at https://osf.io/s52zu/.

## Footnotes

1 Note that this complete 2 × 2 design leads to the fact that one group received the S–E-based instruction to produce a tone, but did not hear any effect tones in the test phase. Pfister et al. (2014) reported congruency effects also when the effects are not perceivable, so we included this group to have a complete, balanced design.

2 Sample size calculation was conducted using *α* = 0.05, power = 90%, *d* = 1.0 for the ideomotor congruency effect (based on pilot studies for this experiment) and *η*_*p*_^2^ = .175 for the main effect of instruction (Eder and Dignath 2017). The two-way ANOVA power analysis revealed a minimal sample size of 18 and 13 for each group of these two factors.

3 As we had no reference effect size for the task relevance and the impact of effect tones in this setting, sample size calculation was conducted using *α* = 0.05, power = 90%, *η*_*p*_² = 0.18 for the ideomotor congruency effect (based on Experiment 1) and revealed a minimum of 13 participants in each group. The experiment consisted of 3 cells in which we compared congruent and incongruent trials, which results in a minimum sample size of *n* = 39.

4 Sample size calculation was conducted using *α* = 0.05, power = 90%, *η*_*p*_² = 0.18 for the ideomotor congruency effect (based on Experiment 1) and revealed a minimum of 13 participants. We chose to test 25 participants in order to reduce the risk of missing a present ideomotor congruency effect.
